# Identification of Medication Prescription Errors and Factors of Clinical Relevance in 314 Hospitalized Patients for Improved Multidimensional Clinical Decision Support Algorithms

**DOI:** 10.3390/jcm12154920

**Published:** 2023-07-26

**Authors:** Stefan Russmann, Fabiana Martinelli, Franziska Jakobs, Manjinder Pannu, David F. Niedrig, Andrea Michelle Burden, Martina Kleber, Markus Béchir

**Affiliations:** 1 Swiss Federal Institute of Technology Zurich (ETHZ), 8093 Zurich, Switzerland; f_martinelli@outlook.de (F.M.); fjakobs@student.ethz.ch (F.J.); andrea.burden@pharma.ethz.ch (A.M.B.); 2 Faculty of Medicine, University of Nicosia, 2408 Egkomi, Cyprus; pannu.man@live.unic.ac.cy (M.P.); markus.bechir@zim.ch (M.B.); 3 Drugsafety.ch, Seestrasse 221, 8703 Küsnacht, Switzerland; david.niedrig@hirslanden.ch; 4 Department of Internal Medicine, Clinic Hirslanden Zurich, 8032 Zurich, Switzerland; martina.kleber@hirslanden.ch; 5 Center for Internal Medicine, Clinic Hirslanden Aarau, 5001 Aarau, Switzerland; 6 Hospital Pharmacy, Clinic Hirslanden Zurich, 8032 Zurich, Switzerland; 7 Faculty of Medicine, University of Basel, Klingelbergstrasse 61, 4056 Basel, Switzerland

**Keywords:** medication errors, adverse drug events, clinical decision support, clinical medicine, clinical pharmacology, drug interactions, dose adjustment, pharmaVISTA, MediQ

## Abstract

Potential medication errors and related adverse drug events (ADE) pose major challenges in clinical medicine. Clinical decision support systems (CDSSs) help identify preventable prescription errors leading to ADEs but are typically characterized by high sensitivity and low specificity, resulting in poor acceptance and alert-overriding. With this cross-sectional study we aimed to analyze CDSS performance, and to identify factors that may increase CDSS specificity. Clinical pharmacology services evaluated current pharmacotherapy of 314 patients during hospitalization across three units of two Swiss tertiary care hospitals. We used two CDSSs (pharmaVISTA and MediQ), primarily for the evaluation of drug-drug interactions (DDI). Additionally, we evaluated potential drug-disease, drug-age, drug-food, and drug-gene interactions. Recommendations for change of therapy were forwarded without delay to treating physicians. Among 314 patients, automated analyses by both CDSSs produced an average of 15.5 alerts per patient. In contrast, additional expert evaluation resulted in only 0.8 recommendations per patient to change pharmacotherapy. For clinical pharmacology experts, co-factors such as comorbidities and laboratory results were decisive for the classification of CDSS alerts as clinically relevant in individual patients in about 70% of all decisions. Such co-factors should therefore be used for the development of multidimensional CDSS alert algorithms with improved specificity. In combination with local expert services, this poses a promising approach to improve drug safety in clinical practice.

## 1. Introduction

Prescription errors and resulting adverse drug events (ADEs) remain a major challenge in clinical medicine, as they increase morbidity, mortality, and healthcare costs [1,2,3,4]. Even though most formal prescription errors may result in no or non-severe reversible ADEs, a considerable absolute number of severe and even fatal cases continue to occur in all health systems despite various countermeasures. The incidence of ADE in inpatients was estimated to be 19%, with a recent study claiming that approximately 64% of ADEs are preventable, which concerns particularly prescription errors [5]. The average cost of an ADE in a hospitalized patient has been estimated at around 2500 Euros, mainly due to a prolonged hospitalization of 1.9 days on average [6,7]. Therefore, ADEs pose not only health risks for patients, but also major economic burden.

Prescription errors often remain undetected in clinical practice, and clinical decision support systems (CDSSs) were developed with the aim to identify prescription errors and prevent resulting ADE [8,9]. CDSSs can sensitively screen for drug interactions but are limited by their low specificity regarding clinical relevance. This causes over-alerting, resulting in an estimated 49–96% of clinicians indiscriminately overriding automated alerts (alert-fatigue) [10,11,12,13,14]. Therefore, the isolated installation of a CDSS in clinical settings has been shown to have only limited efficacy to prevent prescription errors and ADEs [15,16,17,18,19,20]. Most CDSSs and local expert countermeasures primarily focus on drug-drug interactions (DDI). However, the clinical relevance of potential prescription errors is highly complex, as it also relates to indication, dosage, and interactions other than those between two drugs. There are also drug-gene, drug-disease, drug-age, and drug-food interactions, as well as therapeutic duplications. Furthermore, any one potential prescription error may not be sufficient to cause an ADE in an individual patient. Indeed, in patients with a severe ADE, prescription errors and other risk factors typically occur concomitantly as component causes, and their interactions with each other constitute a combined sufficient cause that results in a clinically manifest ADE [21]. This basic concept of disease epidemiology is also applicable to the prevention of ADEs in clinical practice, because it implies that we must look at a range of factors that contribute to the occurrence of clinically manifested ADEs to better understand their causal role, and to improve the specificity and efficacy of CDSS use in clinical practice. Indeed, the identification of multifactorial causes of ADEs and their implementation into CDSS algorithms has been shown to increase specificity and reduce alert fatigue [14,22,23].

Therefore, the purpose of this study was to evaluate clinical pharmacology services that combine the analysis of CDSS alerts with real-time evaluation of other patient-specific factors to provide timely recommendations. Furthermore, we aimed to identify and describe such additional factors used by experts that define the clinical relevance of potential medication errors, because they may be integrated into CDSSs to improve their specificity. Ultimately, our work aims for personalized expert alerts supported by an improved CDSS with higher specificity that have the potential to achieve higher clinical relevance of alerts and more efficient improvement of medication safety in clinical practice.

## 2. Materials and Methods

### 2.1. Study Design, Setting, Patient Population and Procedures

This observational cross-sectional cohort study analyzed routine medication evaluations by clinical pharmacology services of hospitalized patients between February and June 2021 from three different units of two tertiary care hospitals in Switzerland, i.e., two departments of internal medicine (IM1 and IM2), and one emergency room (ER).

Among all patients with at least one drug prescription, we screened all prescriptions of individual patients for potential medication errors within 48 h after hospital admission using a CDSS (whenever clinical pharmacology routine services were available, usually at least 4 days per week). All CDSS alerts were immediately evaluated regarding their clinical relevance for individual patients in the context of their current medical condition by a senior clinical pharmacologist (SR) and a pharmaceutical sciences specialist in training (FM). For that purpose, all available relevant information from the patients’ electronic medical records (EMR) was reviewed, particularly diagnoses, laboratory results, current conditions, and treatment plans. While reviewing the EMR, we also evaluated each patient’s medication regarding potential drug-disease, drug-age, drug-gene and drug-food interactions, dose, therapeutic duplications, and other potential risk factors for ADEs, such as renal impairment, liver disease, or abnormal laboratory results.

Based on the results and conclusions of our evaluations we subsequently provided written specific recommendations for adjustments of the current medication and/or monitoring to the treating physicians on the same day, plus additional direct oral communication whenever required.

### 2.2. Clinical Decision Support Systems (CDSS)

Screening for potential medication errors was performed using two different CDSSs, and we documented all automated alerts generated by the CDSS for each patient.

For IM1 and ER we used pharmaVISTA (https://pharmavista.ch accessed daily between February and June 2021), which was fully integrated into the hospital’s EMR and electronic prescribing (eRx) system. pharmaVISTA provides results in tables, categorized by checks for DDI, therapeutic duplications, excess of maximum dose, drug-age interactions according to Beers’ criteria of potentially inappropriate medication (PIM) for elderly patients [24], and drug-food interactions. pharmaVISTA’s automated check for dose adjustment in renal impairment was not enabled due to the lack of an interface with laboratory results (creatinine/eGFR value required). Therefore, the need for dose adjustment in response to impaired renal function was evaluated manually for all patients by clinical pharmacology services.

For IM2 there was no integrated CDSS available at the time, and we used the online version of the CDSS, mediQ (https://www.mediq.ch (accessed daily between February and June 2021), with manual entry of all prescriptions. MediQ primarily analyzes DDI, and it also provides a general alert if renal or hepatic impairment is relevant for prescribing of specific drugs. If a specific pharmacogenetic variant is known or suspected, this can be manually entered, and MediQ can then also check for drug-gene interactions. This function was not systematically used for all patients, but only on-demand in selected cases. Additional potential issues at IM2 including those analyzed by pharmaVISTA at IM1/ER were evaluated manually by clinical pharmacology services.

### 2.3. Outcomes, Data Analysis, and Ethical Approval

The primary outcome of this analysis was the frequency of automated alerts generated by CDSS compared to the frequency of alerts that were considered as clinically relevant by clinical pharmacology services. The secondary outcome was the qualitative and quantitative description of additional factors that may attenuate the clinical relevance of automated CDSS alerts.

Data analysis was descriptive, with presentation of results in tables and figures as appropriate. For continuous variables with an approximately normal distribution, averages were calculated as arithmetic means, whereas for variables without a normal distribution averages were expressed as median and range. Renal function was categorized based on estimated glomerular filtration rate (eGFR) calculated with the CKD-EPI formula, and according to stages of chronic kidney disease (G1 to G5). Number and distribution of DDI alerts were also presented as box plots showing median, interquartile range and outliers.

We used Microsoft Excel (Redmond, Waukesha, WI, USA) and STATA 17.0 for MacOS (STATA Corp, College Station, TX, USA) for data management, descriptive statistics, and graphic presentation of results.

## 3. Results

### 3.1. Patient Characteristics

The analysis included a total of 314 patients, 153 from IM1, 80 from IM2, and 81 from ER. Their demographics and systematically retrieved laboratory results for serum sodium, potassium and eGFR are shown in Table 1. There was an approximately even sex distribution, and age ranged from 20 to 95 years. Renal function was impaired with an eGFR < 60 mL/min/1.73 m^2^ in 46.2% of patients and <30 mL/min/1.73 m^2^ in 11.6%. Hypo- or hyperkalemia was observed in 6.1% and 4.4%, and hypo- or hypernatremia in 3.3% and 1.3%, respectively.

### 3.2. Drug Prescriptions

Table 2 presents an overview of drug prescriptions in the studied population. Patients had a median number of 7 (range 1–22) drugs with regular daily prescriptions, and 1 (range 0–13) on-demand prescriptions. The three most frequently prescribed drug classes were anticoagulants/antiplatelets, prescribed in 78.0% of patients, followed by antihypertensives and other cardiovascular drugs (67.2%), metamizole and/or paracetamol (57.6%), and proton pump inhibitors (53.8%). Of note, automated duplication alerts were often erroneously generated by the integrated CDSS (pharmaVISTA) when prescriptions were changed from single administration to permanent prescriptions, and therefore, were excluded from our documentation.

### 3.3. Frequency of Potential Medication Errors—CDSS Alerts vs. Expert Recommendations

Table 3a,b show the frequency of potential medication errors (pMEs) according to CDSS alerts versus expert recommendations, stratified over different categories of medication errors. Table 3a expresses the frequency as the percentage of patients with at least one CDSS alert or expert recommendation, and Table 3b as the mean number of alerts or expert recommendations per patient.

The number of automated CDSS alerts was much higher than the number of alerts and recommendations considered clinically relevant by clinical pharmacology experts. CDSSs issued at least one alert for 98.4% of patients, whereas experts eventually issued at least one recommendation in only 36.9% of patients. In other words, the average number of alerts issued by CDSSs was 15.5 per patient, which is much higher than the 0.8 alerts per patient identified by experts.

pharmaVISTA identified potential DDIs for 71.2% and 65.4% at IM1 and ER, respectively, whereas MediQ identified DDI in 100% in IM2. The difference in the average number of issued alerts per patient between the two different CDSSs and sites was even greater, i.e., a mean of 3.0 and 2.5 DDI alerts per patient for pharmaVISTA at IM1 and ER, respectively, vs. 26.2 for MediQ at IM2. Experts issued much fewer recommendations related to potential DDIs, i.e., at least one recommendation in only 16.2% of patients, with a mean of 0.3 per patient. In contrast to CDSS alerts, there were no major differences in the frequency of expert recommendations between IM1/ER and IM2.

Implemented at IM1 and ER, pharmaVISTA also evaluated potential prescription errors related to therapeutic duplications, excess of maximum daily dose, and drug-age and drug-food interactions. Again, Table 3a,b show that the frequency of alerts was much higher for pharmaVISTA compared to issues considered clinically relevant by experts in the current situation of a specific patient. Of note, there was an excessive number of drug-age and drug-food interaction alerts, whereas only a minor fraction of drug-age issues, and none of the drug-food issues appeared actionable.

**Figure 1:**
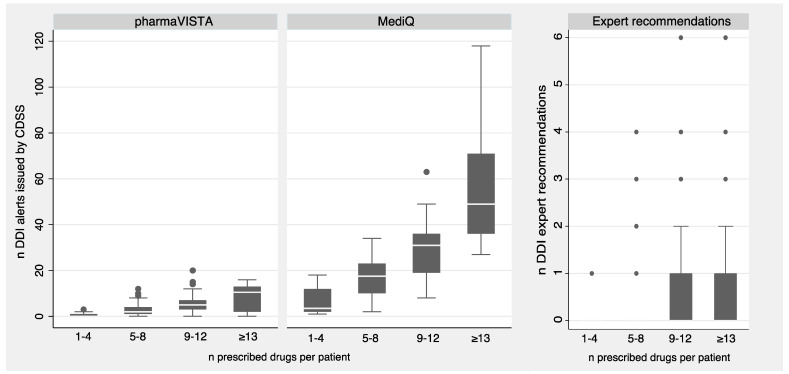
Box plots showing the number of CDSS alerts by pharmaVISTA and MediQ, and expert recommendations for drug-drug interactions, over 4 categories of polypharmacy.

Figure 1 presents box plots of the number of DDI CDSS alerts for pharmaVISTA and MediQ, and for expert recommendations over 4 categories of polypharmacy. The number of CDSS alerts increases with a higher number of prescribed drugs, but this trend is much less pronounced for pharmaVISTA compared to the exponential-like increase seen for MediQ. In contrast, the average number of 0.3 DDI expert recommendations per patient is much lower and does not show a comparable increase if visualized by a box plot.

### 3.4. Specific Potential Medication Errors

Table 4 shows the most frequent potential ADEs that may result from DDIs that had been evaluated as clinically relevant in individual patients, according to experts. QTc prolongation (and consequent increased risk for cardiac arrhythmias) was identified as the main potential ADE in 86 cases, most commonly resulting from a combination of metoclopramide and antipsychotics (n = 19). The second most frequently expected potential ADE (9) was extrapyramidal syndrome (EPS), resulting from the combination of metoclopramide with an antipsychotic, followed by increased drug concentrations due to inhibition of CYP450 enzyme activity with subsequent increased dose-dependent effects (n = 12).

Among all recommendations regarding therapeutic duplications, opioids represented the most frequent drug class, accounting for 13 alerts. This was followed by antihypertensive drugs (n = 5), especially the combination of the two calcium antagonists amlodipine and nifedipine (n = 3).

Medication errors concerning higher-than-recommended doses were most frequently related to higher-than-necessary doses of paracetamol (n = 7), typically 3 × 1000 mg regular prescription instead of sufficient 4 × 500 mg on-demand. Noticeably, this issue was not picked up by either of the CDSSs. Due to admittedly limited acute clinical relevance, however, we forwarded a related recommendation only if additional recommendations in a patient were made. This made the actual frequency of prescribing this unnecessarily high dose much higher than reflected in our recommendations. Most other dosing errors that resulted in recommendations related to an exceeded daily dose (n = 7). Age-related medication errors were mostly caused by either zolpidem or hydrochlorothiazide (n = 5 for each).

### 3.5. Drug-Disease and Drug-Gene Interactions

As part of the expert evaluation process, we considered not only two-factor interactions but also additional co-factors of potential clinical relevance. For example, the decision to issue an expert alert regarding QTc prolongation may depend on the number of concomitantly prescribed drugs causing QT prolongation (>2), their potential to do so, the type of prescription (regular vs. on-demand), and other conditions such as preexisting long QT syndrome. These co-factors were frequently decisive for the classification as clinically relevant, influencing about 70% of all decisions.

All clinically relevant drug-disease interactions are summarized in Table 5. Renal impairment (n = 39) was the most frequent reason for a recommendation regarding a drug-disease complication. Stratifying these patients based on the severity of CKD showed, for example, that 50% of patients with stage 5 kidney disease (eGFR < 15 and/or dialysis) received five recommendations on average. Further drug-disease interactions were mainly related to diabetes (12), osteoporosis (12) and electrolyte imbalances such as hyponatremia (4), hypercalcemia (2) and hyperkalemia (2).

All potential drug-gene interactions that triggered an expert recommendation were related to clopidogrel prescriptions. In 7 out of 22 patients a specific recommendation to perform CYP2C19 pharmacogenetic testing was issued.

## 4. Discussion

This study evaluated the real-life identification of potential prescription errors by clinical pharmacology services using a CDSS. We observed an excessive number of alerts when CDSSs are applied, to analyze real-life polypharmacy in clinical practice, which underlines their overalerting nature and confirms their primary role as screening tools with high sensitivity but low specificity regarding clinical relevance. Furthermore, we found that for clinical pharmacology experts, patient-specific co-factors such as comorbidities and laboratory results were decisive for the classification of CDSS alerts as clinically relevant in about 70% of all decisions. Therefore, the integration of co-factors into CDSS algorithms has the potential to decrease the number of irrelevant alerts and increase their specificity to correct clinically relevant prescription errors, prevent related ADEs, and reduce costs in clinical practice.

Although our results refer only to the described setting, our population of hospitalized patients with high age and a high prevalence of polypharmacy, polymorbidity, and renal impairment can be considered as representative for a typical population of a tertiary care department of internal medicine and an emergency room, respectively. As such, we studied a typical population of inpatients at high risk for an ADE, who are particularly likely to benefit from proactive medication safety checks [25,26,27].

We found a much higher number of DDI alerts from the CDSS at IM1 and ER vs. IM2, but the number of expert alerts were similar for those populations. Therefore, the use of pharmaVISTA at IM1/ER vs. MediQ at IM2 likely explains this observation. pharmaVISTA produces also considerable additional overalerting for drug-food and drug-age interactions. Although drug-age interactions are based on established Beers’ criteria, we found that the majority are clinically irrelevant and, consequently, have little impact in clinical practice. This is in accordance with other recent studies, e.g., Parodi Lòpez et al. reported that only 1 out of 7 potentially inappropriate medications may be clinically relevant, and that among those, half may not be of sufficient priority for medical action [28]. Furthermore, a quantitative comparison of alert frequencies does not necessarily reflect strengths and limitations of a CDSS when they are used in daily routine, where their usability is also determined by the structure and visualization of the presented information. In our experience, for drug safety experts, both CDSSs provide excellent structured summaries in table format at a glance, including categories of potential severity, details on pharmacokinetics and pharmacodynamics, references to related scientific literature, and helpful information that supports clinical decision making. Because the contents of both systems come from different sources, for us, the simultaneous use of both systems for an individual patient as part of a comprehensive evaluation is indeed not unusual.

In contrast, for the average physician, CDSS overalerting is a key factor, resulting in alert-fatigue and compromised real-life efficacy of CDSSs for the prevention of ADEs. With an average number of 12 alerts per patient and the assumption that a physician may simultaneously care for 10–15 patients at a hospital, this would require the evaluation of >120 alerts per day—with only a minority of those being clinically relevant. This process is time-consuming and neither efficient nor effective enough to be integrated into clinical routine. An increase in alert specificity is therefore necessary to limit the number of automated alerts to those that are clinically relevant. Moreover, CDSSs have evolved considerably during the past 10 years, and there appears to be not much room for further improvement if their current principle format remains unchanged. The sole addition of more revised information is unlikely to result in major breakthroughs regarding their impact on drug safety in clinical practice.

One solution for the successful implementation of typical current CDSSs to improve drug safety in hospitals is the involvement of medication safety expert teams that translate CDSS signals to direct care teams of individual patients by carefully evaluating automated alerts in the clinical context of those patients. Indeed, other groups have established expert services for hospitalized patients, where clinical pharmacists use CDSSs as screening tools, and then manually evaluate the large number of alerts according to clinical relevance using additional patient-specific information. Their results are comparable to ours, in the sense that expert services can effectively reduce the number of unnecessary alerts and therefore alert fatigue [12,14,29,30].

Another promising approach is a further development of CDSSs that looks beyond the current focus on just two-dimensional alert algorithms, such as simple two-way drug-drug-interactions. Ideally, a more efficacious CDSS would be inspired by the aforementioned causal pie model [21] and search for clinically relevant component causes of ADEs that can be used for multidimensional alert algorithms. An initial example is the additional implementation of current blood pressure measurements and laboratory results into algorithms. This is expected to markedly increase the specificity of an automated alert, e.g., when a drug-drug interaction increases the risk of hypotension, hyperkalemia, or hyponatremia. Another example that could be implemented into an alert algorithm is a drug-drug-gene interaction [31]. Furthermore, more complex artificial intelligence-based solutions may be able to screen any clinical information documented in a patient’s EMR. Interestingly, recent developments of “next-generation” CDSSs that use artificial intelligence for alert algorithm development also focus on the use patient-specific factors to increase alerts’ specificity, and first evaluations of their performance are promising [23,32,33]. 

In this context, it is of interest that in our analysis, patient-specific co-factors were decisive for the classification of CDSS alerts as clinically relevant in about 70% of all decisions, and that most drug-drug interactions resulting in pADEs are related to electrolyte imbalances as a common root cause. For drug-disease interactions, renal impairment was the most frequently detected cause for expert recommendations, and pharmaVISTA is indeed able to use current serum creatinine results for the estimation of renal function and check for required dose-adjustment. The fact that at our institution, a reliable interface for the use of this function was not yet available demonstrates additional challenges for IT, but with quickly developing hospital IT this should be a solvable problem for health care institutions in the future. Indeed, in a local customized approach, we previously demonstrated the feasibility of this approach > 10 years ago [22]. Therefore, if next generation CDSSs can use patient-specific data, hospital IT must anticipate this development and provide adequate interfaces for the implementation of future CDSSs that will likely overcome important limitations of current CDSSs that are based on relational databases without consideration of patient-specific data.

Finally, we should mention some limitations of our analyses. First, we evaluated only a limited number of patients, whereas a larger sample size would be preferable to define the most important targets for interventions with higher precision, and to better identify related co-factors that attenuate the clinical relevance of medication error alerts. However, we consider our current analysis as a pilot study with the aim to introduce ongoing systematic evaluations of drug safety throughout our network of 17 affiliated hospitals. This approach may allow us to conduct repeated semi-automated analyses of much larger patient numbers in the future. Second, our analysis did not include a control group without immediate interventions through written recommendations to treating physicians, where resulting potential medication errors and adverse events could be analyzed, but this was not possible for obvious ethical reasons. Third, the current project was not able to systematically document the implementation rate of our expert recommendations in all patients. However, our preliminary analysis of patients who we were able to follow-up regarding prescriptions showed an overall implementation rate well above 50%, indicating good efficacy and efficiency of our work. Furthermore, we will collect information on implementation in a subsequent ongoing project and will, therefore, be able to provide more data on implementation rates in the future.

The aim for a high implementation rate of (clinically relevant and, therefore, justified) alerts is indeed a topic that deserves attention and action beyond our study and institution. A strong awareness of the importance of medication error reduction in clinical practice and an ingrained safety culture are paramount and should start with education in medical and pharmacy schools. Furthermore, resources assigned to improving medication safety in hospitals are typically low or even non-existent, which further points to the need for improved specificity of current CDSSs. As mentioned above, future artificial intelligence-based CDSSs that use patient-specific information have the potential to considerably reduce the required resources for drug safety expert hospital pharmacologists and/or pharmacists to implement effective safety measures and, therefore, achieve cost-efficient proactive medication safety management.

## 5. Conclusions

The majority of automated CDSS alerts are clinically irrelevant, which leads to significant over-alerting. Therefore, clinicians tend to generally ignore and override them. This poses an important challenge to the further development of CDSSs. We have successfully identified several potential aspects that could increase CDSS specificity and, thus, its acceptance in clinical practice. Readily available laboratory values and diagnostic codes could be integrated into CDSS algorithms; in particular, lists for inappropriate medication in elderly patients should be revised with the aim of improving currently low clinical relevance. Food alerts should generally not be included in CDSSs, but high-risk foods should rather be avoided in hospital gastronomy altogether. Most important, in a paradigm shift, the development of CDSSs should focus on the further development of two-factorial interactions into multidimensional alert algorithms whenever feasible. Additional variables for such algorithms must be easy to retrieve from EMRs and may include laboratory, imaging, ECG, and other examination results. Even next-generation artificial intelligence-driven multidimensional CDSSs should be combined with proactive programs involving local clinical drug safety experts, and their specificity should be continuously evaluated for further improvement, aiming for higher specificity and efficacy. In such a scenario, the integration of the proposed factors should indeed lead to substantial and cost-efficient improvement of patient safety. Therefore, we are currently working on the systematic development, implementation, and ongoing surveillance of customized multidimensional alert algorithms as part of a closed-loop proactive quality control system for improved medication safety at our hospitals.

## Figures and Tables

**Table 1:** Patient characteristics and basic laboratory values of the study population.

	IM 1		IM 2		ER		Total	
	n	(%)	n	(%)	n	(%)	n	(%)
**Number of patients**	153	(100)	80	(100)	81	(100)	314	100
**Sex**								
Female	68	(44.4)	37	(46.3)	45	(55.6)	150	(47.8)
Male	85	(55.6)	43	(53.7)	36	(44.4)	164	(52.2)
**Age (years)**								
Median (range)	76	(23–93)	75	(36–95)	72	(20–95)	75	(20–95)
Renal function (eGFR, mL/min/1.73 m^2^) *								
≥90	32	(21.5)	14	(18.2)	20	(26.0)	66	(21.8)
60–89	37	(24.8)	31	(40.3)	29	(37.7)	97	(32.0)
30–59	64	(43.0)	17	(22.1)	24	(31.2)	105	(34.7)
15–29	14	(9.4)	13	(16.9)	2	(2.6)	29	(9.6)
<15	2	(1.3)	2	(2.6)	2	(2.6)	6	(2.0)
Potassium **								
Hypokalemia (<3.5 mmol/mL)	13	(8.6)	2	(2.7)	3	(4.2)	18	(6.1)
Hyperkalemia (>5 mmol/mL)	9	(5.9)	1	(1.4)	3	(4.2)	13	(4.4)
Sodium ***								
Hyponatremia (<132 mmmol/mL)	5	(3.3)	2	(2.6)	3	(3.9)	10	(3.3)
Hypernatremia (>146 mmol/mL)	4	(2.6)	0	(0)	0	(0)	4	(1.3)

Missing values for * 11, ** 17 and *** 6 patients, respectively.

**Table 2:** Drug prescriptions of the study population.

	IM 1		IM 2		ER		Total	
	Median	(Range)	Median	(Range)	Median	(Range)	Median	(Range)
Regular prescriptions per patient	7	(1–17)	9	(1–22)	6	(1–16)	7	(1–22)
On-demand prescriptions per patient	0	(0–13)	4	(0–9)	0	(0–8)	1	(0–13)
	**n**	**(%)**	**n**	**(%)**	**n**	**(%)**	**n**	**(%)**
Anticoagulants/antiplatelets	116	(75.8)	70	(87.5)	59	(72.8)	245	(78.0)
Antihypertensives and cardiovascular	104	(68.0)	59	(73.8)	48	(59.3)	211	(67.2)
Metamizole/paracetamol	76	(49.7)	72	(90.0)	33	(40.7)	181	(57.6)
Proton pump inhibitors	78	(51.0)	52	(65.0)	39	(48.2)	169	(53.8)
Opioids	60	(39.2)	39	(48.8)	18	(22.2)	117	(37.3)
Diuretics	46	(30.1)	41	(51.3)	23	(28.4)	110	(35.0)
Cholesterol lowering drugs	50	(32.7)	39	(48.8)	17	(21.0)	106	(33.8)
Antibiotics/antivirals/antifungals	47	(30.7)	26	(32.5)	24	(29.6)	97	(30.9)
Antipsychotics	31	(20.3)	27	(33.8)	7	(8.6)	65	(20.7)
Antidepressants	33	(21.6)	16	(20.0)	13	(16.1)	62	(19.7)
Benzodiazepines/Z-drugs	23	(15.0)	27	(33.8)	9	(11.1)	59	(18.8)
Glucose lowering drugs	21	(13.7)	20	(25.0)	9	(11.1)	50	(15.9)
NSAIDs	9	(5.9)	14	(17.5)	6	(7.4)	46	(14.6)
Hormones	26	(17.0)	5	(6.3)	8	(9.9)	39	(12.4)
Steroids and immunosuppressants	15	(9.8)	11	(13.8)	12	(14.8)	38	(12.1)
Antiasthmatics	17	(11.1)	7	(8.8)	7	(8.6)	31	(9.9)
Antiepileptics	14	(9.2)	8	(10.0)	7	(8.6)	29	(9.2)
Antiarrhytmics	8	(5.2)	6	(7.5)	5	(6.2)	19	(6.1)
Antiparkinson drugs	7	(4.6)	6	(7.5)	5	(6.2)	18	(5.7)
Other	93	(60.8)	79	(98.8)	39	(48.2)	211	(67.2)

**Table 3:** (**a**) Frequency of potential medication errors (pME) expressed as percentage of patients with at least one automated CDSS alert versus patients with expert recommendation, stratified over type of potential error. (IA = interaction). (**b**) Frequency of potential medication errors (pME) expressed as mean number per patient of automated CDSS alerts versus number of expert recommendations, stratified over type of potential error. (IA = interaction).

(a)								
	IM 1 (% of Patients)		IM 2 (% of Patients)		ER (% of Patients)		Total (% of Patients)	
	CDSS pharmaVISTA	Expert	CDSS MediQ	Expert	CDSS pharmaVISTA	Expert	CDSS	Expert
**Drug-Drug IA**	71.2%	15.7%	100%	18.8%	65.4%	14.8%	77.1%	16.2%
**Duplication**	54.3%	7.8%	n.a.	8.8%	49.4%	6.2%	52.6%	7.6%
**Dosage**	11.8%	4.6%	n.a.	5.0%	9.9%	6.2%	11.1%	5.1%
**Drug-Age IA**	75.8%	3.9%	n.a.	3.8%	61.7%	2.5%	70.9%	3.5%
**Drug-Food IA**	98.0%	0%	n.a.	0%	91.4%	0%	95.7%	0%
**Drug-Disease IA**	n.a.	20.3%	n.a.	33.8%	n.a.	17.3%	n.a.	23.1%
**Drug-Gene IA**	n.a.	6.5%	n.a.	7.5%	n.a.	8.1%	n.a.	7.2%
**TOTAL (any pMI)**	**99.4**	**36.6%**	**100**	**48.8%**	**95.1**	**25.9%**	**98.4**	**36.9%**
**(b)**								
	**IM 1 (Mean per Patient)**		**IM 2 (Mean per Patient)**		**ER (Mean per Patient)**		**Total (Mean per Patient)**	
	**CDSS pharmaVISTA**	**Expert**	**CDSS** **MediQ**	**Expert**	**CDSS pharmaVISTA**	**Expert**	**CDSS**	**Expert**
**Drug-Drug IA**	3.0	0.3	26.2	0.4	2.5	0.2	8.8	0.3
**Duplication**	1.2	0.1	n.a.	0.1	1.1	0.1	1.1	0.1
**Dosage**	0.2	0.1	n.a.	0.1	0.1	<0.05	0.1	0.1
**Drug-Age IA**	3.8	<0.05	n.a.	<0.05	2.9	<0.05	3.5	<0.05
**Drug-Food IA**	4.7	0	n.a.	0	3.4	0	4.2	0
**Drug-Disease IA**	n.a.	0.3	n.a.	0.6	n.a.	0.2	n.a.	0.4
**Drug-Gene IA**	n.a.	0.1	n.a.	0.1	n.a.	0.1	n.a.	0.1
**TOTAL (any pMI)**	**12.7**	**0.7**	**26.2**	**1.2**	**10.0**	**0.5**	**15.5**	**0.8**

**Table 4:** Frequency of potential adverse drug effects related to drug-drug interactions with specific examples.

Potential Adverse Effect	n	Example of Drug-Drug Interactions
QTc prolongation with increased risk of cardiac arrhythmia	86	Metoclopramide—antipsychotics Metoclopramide—ondansetron Antidepressant—antidepressant Ondansetron—antipsychotics Ondansetron—amiodarone
Extrapyramidal symptoms (EPS)	19	Metoclopramide—antipsychotics
Increased plasma level via CYP450 inhibition	12	Clarithromycin—trazodone Metoclopramide—trazodone
Reduced absorption	8	Levothyroxin—iron supplements
Hyponatremia	3	Hydrochlorothiazide—antidepressants
Nephrotoxicity	3	Ibuprofen—torasemide Diclofenac—valsartan + hydrochlorothiazide
Decreased plasma level via CYP450 induction/inhibition	3	Triazolam—St. John’s wort Oxcarbazepine—clopidogrel
Increased risk of bleeding	2	Clindamycin—phenprocoumon
Myopathy	2	Atorvastatin—lenalidomide Atorvastatin—amiodarone
Antagonistic effect on dopamine system	2	Metoclopramide—antiparkinson drugs
Electrolyte imbalance	2	Hydrochlorothiazide—lisinopril Digoxin—torasemide
Reduced absorption of vitamin B12	2	Pantoprazole—metformin

**Table 5:** Frequency of disease-related factors that triggered expert recommendations (drug-disease interactions).

n	Risk Factor for Potential Adverse Drug Event	Drugs
39	Renal impairment	Antihypertensives, antidiabetics, NSAID, statins, antibiotics, NOAC, etc.
12	Diabetes	Statins
12	Osteoporosis	Vit.D and calcium containing supplements
10	Hepatotoxicity	NSAIDs, statins, neuroleptics, antibodies, antibiotics
6	Heart disease	NSAIDs, antidepressants
6	Hypotension	Antihypertensives
4	Hyponatremia	Antihypertensives, particularly diuretics
4	Urinary retention	Opioids, drugs for gastrointestinal symptoms
3	Infectious disease	Antibiotics, antidiabetics
2	Fall	Benzodiazepines, antiepileptics
2	Hypercalcemia	Vit.D and calcium containing supplements
2	Hyperkalemia	Antihypertensives
10	Other	Opioids, benzodiazepines, antihistamines, hormones

## Data Availability

The data that support the findings of this study are available on request from the corresponding author. The data are not publicly available due to privacy or ethical restrictions.
